# Brain Structural Network Compensation Is Associated With Cognitive Impairment and Alzheimer’s Disease Pathology

**DOI:** 10.3389/fnins.2021.630278

**Published:** 2021-02-25

**Authors:** Xiaoning Sheng, Haifeng Chen, Pengfei Shao, Ruomeng Qin, Hui Zhao, Yun Xu, Feng Bai, the Alzheimer’s Disease Neuroimaging Initiative

**Affiliations:** ^1^Department of Neurology, Affiliated Drum Tower Hospital of Medical School and The State Key Laboratory of Pharmaceutical Biotechnology, Institute of Brain Science, Nanjing University, Nanjing, China; ^2^Jiangsu Province Stroke Center for Diagnosis and Therapy, Nanjing, China; ^3^Nanjing Neuropsychiatry Clinic Medical Center, Nanjing, China

**Keywords:** cognitive impairment, pathological biomarkers, Alzheimer’s disease, structural compensation ability, gray matter (GM) atrophy

## Abstract

**Background:**

Structural network alterations in Alzheimer’s disease (AD) are related to worse cognitive impairment. The aim of this study was to quantify the alterations in gray matter associated with impaired cognition and their pathological biomarkers in AD-spectrum patients.

**Methods:**

We extracted gray matter networks from 3D-T1 magnetic resonance imaging scans, and a graph theory analysis was used to explore alterations in the network metrics in 34 healthy controls, 70 mild cognitive impairment (MCI) patients, and 40 AD patients. Spearman correlation analysis was computed to investigate the relationships among network properties, neuropsychological performance, and cerebrospinal fluid pathological biomarkers (i.e., Aβ, t-tau, and p-tau) in these subjects.

**Results:**

AD-spectrum individuals demonstrated higher nodal properties and edge properties associated with impaired memory function, and lower amyloid-β or higher tau levels than the controls. Furthermore, these compensations at the brain regional level in AD-spectrum patients were mainly in the medial temporal lobe; however, the compensation at the whole-brain network level gradually extended from the frontal lobe to become widely distributed throughout the cortex with the progression of AD.

**Conclusion:**

The findings provide insight into the alterations in the gray matter network related to impaired cognition and pathological biomarkers in the progression of AD. The possibility of compensation was detected in the structural networks in AD-spectrum patients; the compensatory patterns at regional and whole-brain levels were different and the clinical significance was highlighted.

## Introduction

Alzheimer’s disease (AD), the most prevalent cause of dementia, is characterized by progressive loss in the activities of daily living and cognitive impairment, which causes a very large socioeconomic burden (van der Lee et al., 2018). The number of individuals with AD is increasing significantly every year, and 10–20% of people aged 65 or older suffer from mild cognitive impairment (MCI) which is known as a prodromal clinical stage of AD (Kim et al., 2020). However, the effective period of symptomatic treatment is limited (Patnode et al., 2020). Therefore, the early diagnosis and prognosis of clinical AD-spectrum patients is of great importance as it increases the time window to implement interventions that attenuate or reverse deterioration (Luo et al., 2019).

Structural magnetic resonance imaging (MRI) is a promising approach used to identify the progression of disease (Lane et al., 2019). Evidence has been accumulating that changes leading to cognitive impairment and dementia are not limited to specific regions but rather exhibit widespread changes in connectivity and topological properties that have emerged as potential intermediate biomarkers for AD (Verfaillie et al., 2018). The pattern of gray matter morphology can be defined as a network that consists of multiple regions (i.e., nodes) that are interconnected when structural similarity is exhibited within the cortex across subjects (Beheshti et al., 2017). The advantage of examining the morphology of gray matter networks is that it provides the possibility to accurately quantify individual brains using tools from graph theory (Batalle et al., 2013; Beheshti et al., 2017). For example, the small world coefficient provides an indication of whether the organization of connections in the network is different from a randomly organized network (Zhao et al., 2017). Although the biological significance of structural similarities is not fully understood, the similarity within gray matter has been shown to be related to synchronized maturation between brain regions, which may reflect a higher degree of clustering (Wang et al., 2018). Previous studies have shown that changes in structural properties in gray matter are related to the degree of cognitive impairment and disease severity in individuals with AD (Vipin et al., 2018). In the early and preclinical stages of dementia, the gray matter network might commence reorganization and show high resilience to network integrity damages (Lin et al., 2020). Previous studies have further demonstrated that lower cerebrospinal fluid (CSF) Aβ42 levels in individuals with cognitive impairment were closely associated with the perceived decline in memory performance (Zhang et al., 2018). In a series of structural neuroimaging studies, it was reported that individuals with cognitive impairment exhibited, from the perspective of topological properties, higher nodal degree centrality and lower nodal betweenness in the bilateral hippocampus, compared to the healthy controls (Chen et al., 2020). Recently, structural similarity within the gray matter network in individuals with cognitive impairment was mainly related to the thalamus, insula, and occipital cortex and was associated with poor memory performance (Ahmed et al., 2019). However, there has been no research exploring the altered structural network measures related to pathological biomarkers in combination with the structural similarity and topological properties in patients with cognitive impairment. If individuals with cognitive impairment at the early stage of AD could be identified, they may benefit from early targeted intervention. With developments in neuroimaging, an increasing number of studies have focused on identifying brain functional and structural alterations related to the AD continuum, which may potentially be considered a biomarker of AD pathology.

To this end, we compared the structural networks and the structural similarity within gray matter in AD-spectrum patients using a graph theoretical approach (Rubinov and Sporns, 2010). In the present study, the aim was to explore whether gray matter network parameters were linked to declines in cognitive impairment and abnormal CSF pathology in AD-spectrum patients.

## Materials and Methods

Data used in the preparation of our study were obtained from the Alzheimer’s Disease Neuroimaging Initiative (ADNI) database^1^. The protocol was authorized by the ADNI and informed consent was obtained according to the Declaration of Helsinki. The ADNI was launched in 2003 as a non-profit organization, led by Principal Investigator Michael W. Weiner, MD. The aim of the ADNI is to test whether neuroimaging, neuropsychological assessment, and biological markers could track the progression of AD and conduct early diagnosis. For up-to-date information, see adni-info.org.

### Study Population

This study included 34 healthy controls (HC), 70 early or late MCI patients, and 40 AD patients, and used a subset of T1-weighted MRI images for these 144 subjects. Subjects were originally recruited for ADNI-GO or ADNI-2. Group inclusion criteria were as follows. HC subjects had no memory complaints, a CDR score of 0 and Mini-Mental State Examination (MMSE) scores between 26 and 30. MCI subjects had a CDR score of 0.5, MMSE scores between 21 and 30, as well as memory complaints and abnormal memory function according to the Logical Memory II subscale (Delayed Paragraph Recall) from the Weschler Memory Scaled—Revised (=8 for 16 years and more of education; =4 for 8–15 years of education; and =2 for 0–7 years of education), but an absence of dementia. To be included in the AD group, participants had memory complaints, CDR scores between 0.5 and 2.0, MMSE scores less than 26, and presented the criteria for probable AD diagnosis according to National Institute of Neurological and Communicative Disorders and Stroke/Alzheimer’s Disease and Related Disorders Association (NINCDS/ADRDA) (Lu et al., 2019). In addition, we also excluded participants with a history of significant psychiatric and neurological illness (e.g., depression, stroke, traumatic brain injury, and others). All participants were required to provide informed consent compatible with the local sites (Institutional Review Board regulations). Table 1 shows the detailed clinical and demographic information for these subjects.

**TABLE 1:** Demographic and neuropsychological data.

**Items**	**HC (*n* = 34)**	**MCI (*n* = 70)**	**AD (*n* = 40)**	**F/χ 2**	***P***
**Demographics**					
Age (years)	72.380.87	73.780.84	74.851.37	1.082	0.342^*b*^
Education (years)	16.210.48	15.730.34	15.150.45	1.273	0.283^*b*^
Gender (male/female)	13/21	42/28	23/17	4.614	0.100^*a*^
**General cognition**					
MMSE	28.760.26	27.610.21	23.250.30	9.653	<0.001^b^*
MoCA	25.850.40	22.440.34	17.280.68	61.959	<0.001^b^*
FAQ	0.410.24	2.770.37	15.11.06	143.618	<0.001^b^*
CDRSB	0.150.07	1.660.10	5.030.22	268.077	<0.001^b^*
ADAS13	8.090.68	17.30.83	30.681.35	102.456	<0.001^b^*
EcogSPMem	1.470.11	2.210.09	3.250.10	63.732	<0.001^b^*
EcogSPLang	1.330.09	1.560.07	2.470.13	36.699	<0.001^b^*
EcogSPVisspat	1.140.05	1.420.06	2.600.15	61.820	<0.001^b^*
EcogSPPlan	1.300.09	1.490.07	2.760.13	63.467	<0.001^b^*
EcogSPOrgan	1.380.11	1.640.08	3.030.12	71.906	<0.001^b^*
EcogSPDivatt	1.440.11	1.850.09	3.090.13	54.816	<0.001^b^*
EcogSPTotal	1.350.08	1.700.06	2.830.10	78.533	<0.001^b^*
**Cerebrospinal fluid**					
Aβ (pg/mL)	1293.7384.83	901.0557.21	647.9841.89	23.100	<0.001^b^*
t-tau (pg/mL)	210.0313.12	290.8322.36	358.1626.69	7.873	0.001^b^*
p-tau (pg/mL)	19.321.24	28.252.45	35.432.81	7.913	0.001^b^*

Values are presented as the mean ± standard error (SE).^*a*^The *p-*value was obtained by χ^2^ test, ^*b*^the *p-*value was obtained by one-way ANOVA.*Indicates a statistical difference between groups, *p* < 0.05.MMSE, mini mental state examination; MoCA, Montreal Cognitive Assessment; FAQ, Functional Activities Questionnaire; CDRSB, Clinical Dementia Rating Sum of Boxes; ADAS13, Alzheimer’s Disease Assessment Scale; EcogSP, Everyday Cognition by the patient’s study; Mem, Memory; Lang, Language; Visspat, Visuospatial; Plan, Planning; Organ, Organization; Divatt, Divided Attention; Aβ, amyloid-β; t-tau, total tau; p-tau, phosphorylated tau.

### Clinical and Neuropsychological Measurement

All participants received a series of cognitive evaluations in the primary analyses, including the MMSE, Montreal Cognitive Assessment (MoCA); Functional Activities Questionnaire (FAQ); Clinical Dementia Rating Sum of Boxes (CDRSB); Alzheimer’s Disease Assessment Scale (ADAS13), and Everyday Cognition by the patient’s study partner (EcogSP), that provided memory, language, visuospatial abilities, planning, organization, divided attention, and total scores (Table 1).

### Cerebrospinal Fluid Biomarkers

Lumbar puncture and the preparation of the CSF sample were described in the ADNI manual^2^. CSF Aβ, t-tau, and p-tau were measured based on the reagents (Innotest, Fujirebio, Ghent, Belgium) from INNOBIA AlzBio3 immunoassay kit. Not all subjects had CSF sample data because lumbar puncture is an invasive procedure. In this study, 23 out of 34 HC subjects, 46 out of 70 MCI subjects, and 34 out of 40 AD subjects had a CSF sample available (Table 1).

### MRI Acquisition

**FIGURE 1:**
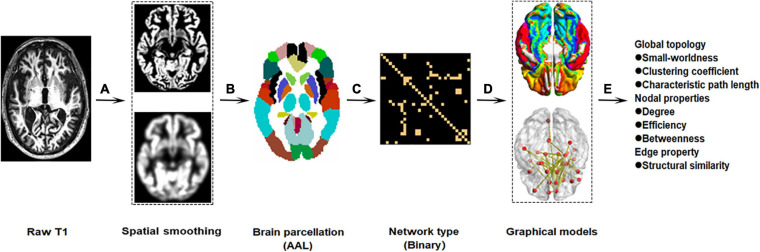
Workflow graphic of gray matter structural networks. The processing of gray matter structural network roughly includes preprocessing (**A:** spatial smoothing), brain parcelation **(B)**, network type **(C)**, graphical models **(D)**, and network reconstruction **(E)**.

The standardized T1-weighted image protocol used volumetric 3-dimensional sagittal MPRAGE^3^. Briefly, the ADNI protocol includes T1-weighted acquisition based on a sagittal volumetric magnetization-prepared rapid gradient-echo sequence collected from a variety of 3.0 Tesla MRI systems with protocols optimized for each type of scanner. Representative of each scan, parameters were as follows: repetition time = 2300 ms; flip angle = 8°; inversion time = 1000 ms; field of view = 240 mm × 240 mm; and a 256 × 256 matrix yielding, a voxel size of 0.94 mm × 0.94 mm × 1.2 mm. The workflow graphic about the processing of the gray matter structural network is presented in Figure 1.

### Image Pre-processing

We used the Computational Anatomy Toolbox (CAT12^4^) as implemented in the Statistical Parametric Mapping analysis package (SPM12^5^) to pre-process the structural images. First, the raw MRI data were checked manually to ensure no obvious artifacts. Second, individual 3D-T1 images were segmented into white matter (WM), gray matter (GM), and cerebrospinal fluid (CSF) using an adaptive Maximum A Posterior technique (Wang et al., 2016). The intracranial volume was obtained by summing the volumes of the GM, WM, and CSF. Last, the resultant GM images were normalized to the Montreal Neurological Institute (MNI) space and the GM volume maps were smoothed spatially (Gaussian kernel of 8 mm full width at half maximum). To define the network nodes, an automated anatomical labeling (AAL) atlas was used to divide the brain into 90 regions of interest (ROIs) (abbreviations provided in Supplementary Table 1). We calculated the gray matter network density considered as the total number of edges in the network, divided by the possible number of edges, and average network strength considered as the sum of all weighted edges for every node, using the Graph Theoretical Network Analysis Toolbox (GRETNA^6^) based on Brain Connectivity Toolbox (Wang et al., 2015).

#### Network Parameters and Network Reconstruction

Every subject’s gray matter network from gray matter segmentations was extracted, using a fully automated method to implement in MATLAB^7^. Briefly, we defined nodes as 3 × 3 × 3 voxel regions in gray matter through an atlas free approach (Rimkus et al., 2019). We then defined connectivity using statistical similarity in gray matter structures by Spearman’s correlations across intensity values of corresponding voxels between one node and neighbor nodes in the gray matter (Tijms et al., 2016). All similarity values were collected in a matrix. Nodes connected were ensured that all subjects had a threshold that they had a similar chance including at most 5% spurious connections through a random permutation method (Toppi et al., 2012). To reduce the number of local tests, the nodal network characteristics for nodes were averaged in 90 regions of interest as defined by the automatic anatomical labeling (AAL) brain atlas (Tzourio-Mazoyer et al., 2002; Jin et al., 2020). The network metrics were classified as “basic” or “higher-order” parameters (Liu et al., 2020). The basic parameters included the local and global degree and the small-worldness. Higher-order network parameters consisted of the clustering coefficient, characteristic path length, degree centrality, and betweenness centrality (Rubinov and Sporns, 2010). To further explore the topological structure of the network, we calculated the small-worldness, global efficiency, and local efficiency. To obtain the network edge, we calculated the connectivity referred to the statistical similarity between each pair of 90 ROIs, which is computed by the Spearman’s correlation of the grey matter intensity values of the corresponding voxels in the ROIs. All similarity values are arranged in a similarity matrix. ROIs are connected when the similarity value of ROIs exceeds the statistical threshold (*P* < 0.05, False Discovery Rate, FDR corrected) determined by the random arrangement method (Toppi et al., 2012). A brief description of specific definitions, calculating formula, and topological property descriptions for the network G with N nodes and V edges follows below (Xu et al., 2016; Yang et al., 2020).

#### Global Topological Properties

The inverse of the harmonic mean of the shortest path length between every two nodes in the network is considered as Global efficiency. It efficiently measures the information communication capacity of the whole network. It is calculated as:

$$E\,g\,l\,o\,b\,a\,l\,\left(G\right)=\frac{1}{N\,\left(N-1\right)}\,\sum_{i\neq j\in G}\frac{1}{d\,i\,j}$$

*d*_*ij*_ is the shortest path length between node i and j of the network.

Local efficiency of the network measures how efficiently the communication information is among the neighbors of a specific node when that node is removed, which shows how fault tolerant the network is and is calculated as:

$$E\,l\,o\,c\,a\,l\,\left(G\right)=\frac{1}{N}\,\sum_{i\in G}E\,g\,l\,o\,b\,a\,l\,\left(G\,i\right)$$

*G*_*i*_ is the subgraph consisting of the nearest neighbors of node *i*.

#### Nodal Topological Properties

Nodal global efficiency quantifies how efficiently the parallel information transfers from one node in the network and is calculated as:

$$E\,n\,o\,d\,a\,l\,\_\,g\,l\,o\,b\,a\,l\,\left(i\right)=\frac{1}{N-1}\,\sum_{i\neq j\in G}\frac{1}{d\,i\,j}$$

*d*_*ij*_ indicates the shortest path length between node i and j of the network.

Nodal local efficiency indicates the efficiency of the communication among the first neighbors of one node when it is removed. It is calculated as:

$$E_{n\,o\,d\,a\,l\,\_\,l\,o\,c\,a\,l}\,\left(i\right)=\frac{1}{N_{i}\,\left(N_{i}-1\right)}\,\sum_{m\neq n\in G_{i}}\frac{1}{d_{m\,n}}$$

G_*i*_ is the sub-graph which consists of node i and its local neighbors.

Nodal strength is defined as the sum of the edge weights in a subnetwork G_*i*_, which is the graph that includes the nodes that are direct neighbors of node i. It can be defined as:

$$S_{n\,o\,d\,a\,i}\,\left(i\right)=\sum_{j\in G_{i}}w_{i\,j}$$

where w_*ij*_ is the edge weight linking node i and j in the subnetwork G_*i*_.

### Statistical Analysis

Statistical analyses were performed with the Statistical Package for the Social Sciences (SPSS, IBM v22). A one-way analysis of variance (ANOVA) was performed in the analyses of age, education, and data volume, with significance at *P* < 0.05 among the control group, the MCI group, and the AD group. The Chi-squared (χ^2^) test was applied in the analysis of gender, among the three groups. Because the neuropsychological data was of non-normal distribution, the Kruskal–Wallis test was applied in the analyses of the neuropsychological data with significance at *P* < 0.05 among the three groups (Zhu et al., 2016).

At the level of the edge properties of the brain network, we used the two-sample t test to investigate group differences between any two groups, adjusting for age, sex, and education years with a false discovery rate (FDR) correction for multiple comparisons.

One-way analysis of covariance (ANCOVA) was used to explore the group differences in the structural networks (degree centrality, betweenness centrality, global efficiency, and local efficiency) while adjusting based on age, sex, and education years. Correction of multiple testing used the FDR. Subsequently, we conducted a *post hoc* analysis to investigate the group differences between any pair of all groups.

Additionally, a multiple linear regression analysis was conducted to investigate the relationships among CSF pathology indicators, gray matter network graph theoretical properties, and cognitive function adjusting for age, gender, and education years at *P* < 0.05, uncorrected (Lu et al., 2017; Wang et al., 2020).

## Results

### Demographic, Neuropsychological, and CSF Data

The characteristic demographic, neuropsychological and CSF data of the participants are presented in Table 1. No significant differences among the three groups were observed in age, gender, or education years (*P* > 0.05). Multiple cognitive functions were more impaired in MCI and AD patients than in the controls, and the largest differences were between AD patients and the controls (all *P* < 0.05), including scores on the MMSE, MoCA, FAQ, CDRSB, ADAS13, and EcogSP.

We observed a significant reduction in CSF Aβ levels (*P* < 0.001) and increased CSF t-tau (*P* = 0.001) and p-tau (*P* = 0.001) levels with the progression of AD.

### Global Topology of Gray Matter Structural Networks

**FIGURE 2:**
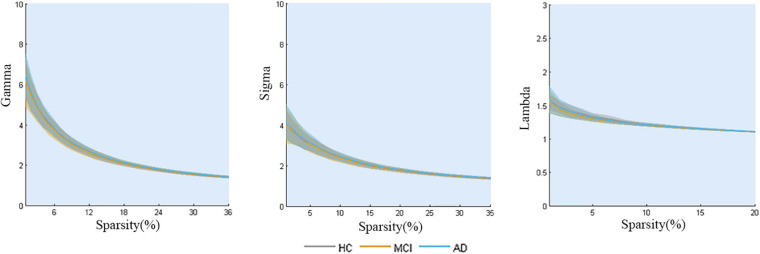
Global topology of gray matter structural networks in AD-spectrum patients. No significant differences were calculated among the three groups (all *P* > 0.05, FDR corrected).

The properties of the global network analysis are shown in Figure 2. No significant differences were calculated among the three groups in global efficiency or the small-worldness (*P* > 0.05, FDR corrected).

### Node-Based Analysis of Gray Matter Structural Networks

**FIGURE 3:**
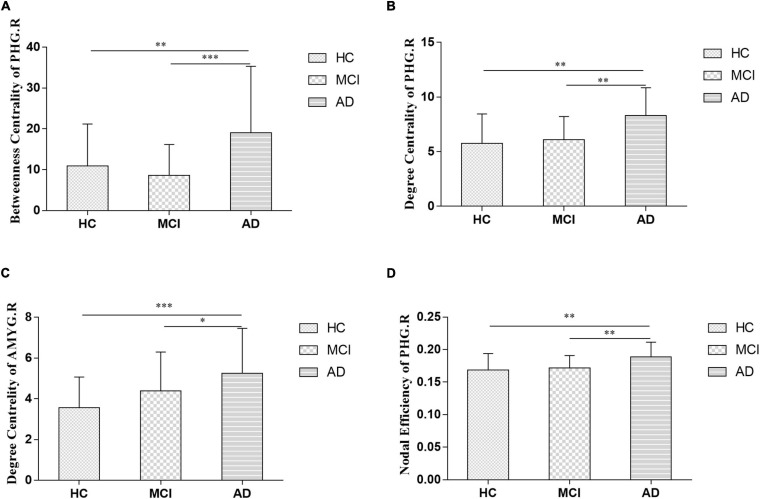
Between-groups comparisons showed the altered betweenness centrality (**A:** right parahippocampal gyrus), degree centrality (**B:** right parahippocampal gyrus; **C:** right amygdala) and nodal efficiency (**D:** right parahippocampal gyrus) in AD-spectrum patients. PHG.R, right parahippocampal gyrus; AMYG.R, right amygdala; *P < 0.05, **P < 0.01, ***P < 0.001 indicates a statistical difference between groups by FDR corrected.

The nodal analysis is shown in Figure 3. Abnormal nodal levels (betweenness centrality, degree centrality, and nodal efficiency) were observed in AD-spectrum patients (*P* < 0.05, FDR corrected). In general, gradually increasing nodal properties in the medial temporal lobe (right parahippocampal gyrus and right amygdala) were associated with the progression of AD across the three groups (from HC to MCI to AD), with the exception of decreased betweenness centrality of the right parahippocampal gyrus in the MCI group.

**FIGURE 4:**
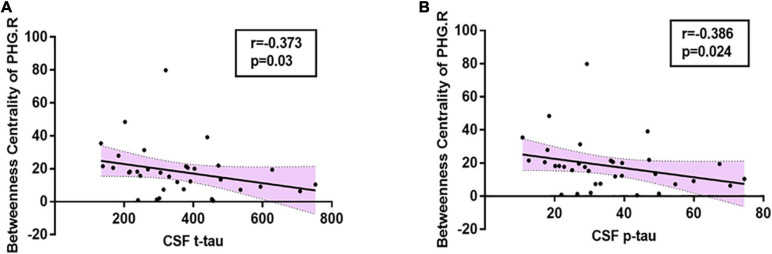
Relationships between altered nodal properties and CSF biomarkers in AD group. The betweenness centrality in the right parahippocampal gyrus was negatively correlated with CSF t-tau (**A:** *r* = -0.373, *P* = 0.03) and CSF p-tau (**B:** *r* = -0.386, *P* = 0.024) in AD patients. PHG.R, right parahippocampal gyrus.

In the present study, significant relationships between altered nodal (i.e., right parahippocampal gyrus and right amygdala) properties and multidomain cognitive impairments were observed in AD-spectrum patients (Table 2; for more details, see Supplementary Table 2). In addition, no significant correlation was calculated between altered nodal properties and CSF biomarkers in HC and MCI patients, and the betweenness centrality in the right parahippocampal gyrus was negatively correlated with CSF t-tau (*r* =-0.373, *P* = 0.03) (Figure 4A) and p-tau (*r* = -0.386, *P* = 0.024) (Figure 4B) concentration in AD patients.

**TABLE 2:** Significant correlations between altered graph theoretical properties and neuropsychological performance in AD-spectrum patients.

**Neuropsychological scale**	**Group**	**Network properties**	**Spearman’s correlation coefficient**	***P-*values**
FAQ	HC	DC of PHG.R	0.352	0.041*
		DC of AMYG.R	0.36	0.037*
		NE of PHG.R	0.362	0.035*
	MCI	BC of PHG.R	0.301	0.011*
	AD	DC of PHG.R	0.334	0.035*
		DC of AMYG.R	0.335	0.034*
		NE of PHG.R	0.342	0.031*
CDRSB	HC	BC of PHG.R	0.37	0.031*
		DC of PHG.R	0.424	0.012*
		DC of AMYG.R	0.388	0.023*
		NE of PHG.R	0.433	0.011*
	MCI	BC of PHG.R	0.295	0.013*
		DC of PHG.R	0.343	0.004**
		NE of PHG.R	0.286	0.016*
	AD	DC of PHG.R	0.339	0.032*
		NE of PHG.R	0.362	0.022*
ADAS13	AD	DC of PHG.R	0.395	0.012*
		NE of PHG.R	0.364	0.021*
EcogSP Mem	MCI	DC of AMYG.R	–0.236	0.049*
EcogSP Lang	HC	BC of PHG.R	0.392	0.022*
	MCI	NE of PHG.R	0.248	0.038*
EcogSP Visspat	HC	BC of PHG.R	0.355	0.039*
		DC of PHG.R	0.492	0.003**
		NE of PHG.R	0.477	0.004**
	MCI	DC of AMYG.R	–0.244	0.041*
	AD	BC of PHG.R	0.334	0.035*
		DC of PHG.R	0.462	0.003**
		DC of AMYG.R	0.384	0.014*
		NE of PHG.R	0.511	0.001**
EcogSP Plan	HC	BC of PHG.R	0.386	0.024*
		DC of PHG.R	0.436	0.01**
		NE of PHG.R	0.454	0.007**
	MCI	BC of PHG.R	0.326	0.006**
	AD	DC of AMYG.R	0.395	0.012*
		NE of PHG.R	0.336	0.034*
EcogSP Organ	HC	BC of PHG.R	0.464	0.006**
		DC of PHG.R	0.484	0.004**
		NE of PHG.R	0.494	0.003**
EcogSP Divatt	HC	BC of PHG.R	0.348	0.044*
		DC of PHG.R	0.491	0.003**
		NE of PHG.R	0.498	0.003**
	AD	NE of PHG.R	0.328	0.039*
EcogSP Total	HC	BC of PHG.R	0.394	0.021*
		DC of PHG.R	0.4	0.019*
		NE of PHG.R	0.413	0.015*
	AD	DC of PHG.R	0.407	0.009**
		DC of AMYG.R	0.389	0.013*
		NE of PHG.R	0.446	0.004**

*P < 0.05, **P < 0.01 indicates an uncorrected relevant analysis.HC, healthy controls; MCI, mild cognitive impairment; AD, Alzheimer’s disease; BC, Betweenness Centrality; DC, Degree Centrality; NE, Nodal Efficiency; PHG.R, right parahippocampal gyrus; AMYG.R, right amygdala; MMSE, mini mental state examination; MoCA, Montreal Cognitive Assessment; FAQ, Functional Activities Questionnaire; CDRSB, Clinical Dementia Rating Sum of Boxes; ADAS13, Alzheimer’s Disease Assessment Scale; EcogSP, Everyday Cognition by the patient’s study; Mem, Memory; Lang, Language; Visspat, Visuospatial; Plan, Planning; Organ, Organization; Divatt, Divided Attention.

### Connectivity-Based Analysis

**FIGURE 5:**
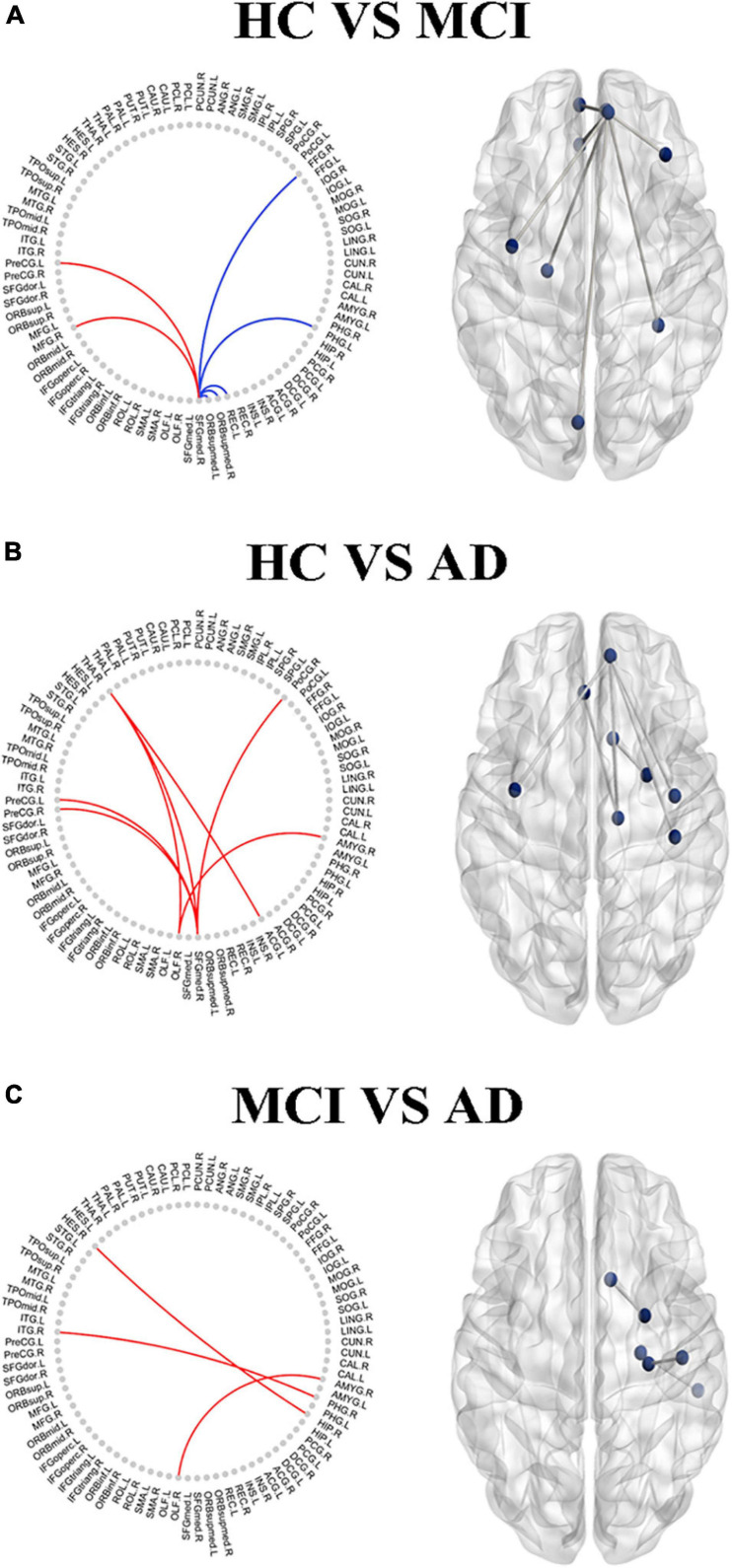
The altered edge based on the node analysis in AD-spectrum patients. Edges with significant (*P* < 0.05, FDR correction) increase (in red) or decrease (in blue) in MCI **(A)** and AD **(B)** in patients compared with HC, and MCI group compared with AD **(C)**. Results are shown in anatomical view (left panels) and in connectograms (right panels).

By using correcting for multiple comparisons with FDR correction, the AD-spectrum patients had significant differences in the structural similarity within the gray matter network when compared to the controls. In addition to a few edges showing decreased structural similarity, most of the others showed increases with the development of AD. In detail, the abnormal connections were mainly related to the frontal lobe in the MCI group (Figure 5A), but were more widely distributed in the frontal lobe, thalamus, and subcortical structures in the AD group (Figures 5B,C) (*P* < 0.05, FDR corrected).

**FIGURE 6:**
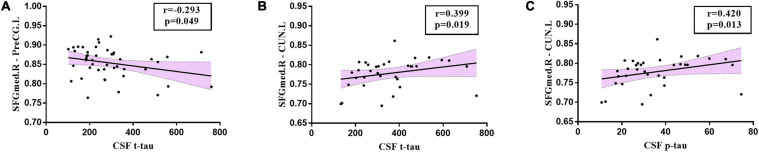
Relationships between altered edges and CSF biomarkers in MCI and AD group. In MCI group, **(A)** the connection between right medial superior frontal gyrus and left precentral gyrus (t-tau: *r* = -0.293, *P* = 0.049) was correlated with CSF tau concentration in MCI patients. In the AD group, the connection between right medial superior frontal gyrus and left cuneus was positively correlated with CSF t-tau (**B:** *r* = 0.399, *P* = 0.019) and CSF p-tau (**C:** *r* = 0.420, *P* = 0.013). SFGmed.R, right medial superior frontal gyrus; PreCG.L, left precentral gyrus; CUN.L, left cuneus.

Significant associations between altered edge properties and cognitive impairments were detected in AD-spectrum patients. Interestingly, most of these connections between edges were associated with the frontal lobe in both the MCI and AD groups (Table 3, for more details, see Supplementary Tables 3, 4). In addition, the connection between the right medial superior frontal gyrus and left precentral gyrus (t-tau: *r* = -0.293, *P* = 0.049) (Figure 6A) was negatively correlated with the CSF tau concentrations in MCI patients. In the AD group, the connection between the right medial superior frontal gyrus and left cuneus was positively correlated with CSF t-tau (*r* = 0.399, *P* = 0.019) (Figure 6B) and CSF p-tau (*r* = 0.420, *P* = 0.013) (Figure 6C).

**TABLE 3:** Significant correlations between the altered edge properties and neuropsychological performance in MCI and AD patients.

**Neuropsychological scale**	**Group**	**Edge**	**Spearman’s correlation coefficient**	***P−*values**
MMSE	MCI	SFGmed.R – MFG.R	0.356	0.002**
		SFGmed.R – ORBsupmed.L	–0.277	0.02*
	AD	SFGmed.R – PreCG.R	–0.396	0.011*
MoCA	AD	SFGmed.R – PreCG.R	–0.35	0.027*
		SFGmed.R – PoCG.R	–0.395	0.012*
		ACG.L – THA.R	–0.349	0.027*
FAQ	AD	SFGmed.R – PreCG.R	0.385	0.014*
CDRSB	AD	SFGmed.R – PoCG.R	0.315	0.048*
ADAS13	AD	ACG.L – THA.R	0.354	0.025*
EcogSP Lang	AD	SFGmed.R – ORBsupmed.R	0.319	0.045*
EcogSP Plan	MCI	SFGmed.R – FFG.R	0.308	0.009**

*P<0.05, **P<0.01 indicates an uncorrected relevant analysis.MCI, mild cognitive impairment; AD, Alzheimer’s disease; MMSE, mini mental state examination; MoCA, Montreal Cognitive Assessment; FAQ, Functional Activities Questionnaire; CDRSB, Clinical Dementia Rating Sum of Boxes; ADAS13, Alzheimer’s Disease Assessment Scale; EcogSP, Everyday Cognition by the patient’s study; Lang, Language; Plan, Planning; PreCG.R, right precentral gyrus; MFG.R, right middle frontal gyrus; SFGmed.R, right superior frontal gyrus-medial part; ORBsupmed.L, left superior frontal gyrus-medial orbital part; ORBsupmed.R, right superior frontal gyrus-medial orbital part; THA.R, right thalamus; PoCG.R, right postcentral gyrus; ACG.L, left anterior cingulate and paracingulate gyri; FFG.R, right fusiform gyrus.

## Discussion

In the present study, we investigated topological alterations in the structural network within gray matter, the relationships to pathological biomarkers, and their behavioral significance in AD-spectrum patients. The three main findings are as follows: (i) The local regional rearrangements in AD-spectrum patients are mainly in the medial temporal lobe. (ii) The rearrangements in the whole-brain networks gradually extended from the frontal lobe to become widely distributed in the cortex with the progression of AD. (iii) These rearrangements in gray matter might be associated with compensation, which was influenced following multidomain cognitive impairments and AD-related CSF.

Research interest is transforming to increasingly earlier diagnoses, since the origin of AD and the key to treatment probably lie in preventing progression to a fully-fledged disease (Hem et al., 2016; Slot et al., 2018). It should be noted that compensation in the structural network has been shown to be manifested earlier in AD-spectrum patients (Liu et al., 2020), and there is increasing interest in the study of structural network alterations to assess the progression in subjects who have a higher risk of AD (Sanchez-Benavides et al., 2018). Therefore, it is essential to evaluate alterations in structural networks related to cognition and pathology (Dicks et al., 2020). In the present study, we deduced that there was the possibility of compensation in the structural networks in AD-spectrum patients, as expected from previous AD-spectrum studies which also provided additional evidence for our research results (Wook Yoo et al., 2015; Caso et al., 2016). Our findings demonstrate that brain regional compensation may start from the medial temporal lobe, and the level of compensation within the whole gray matter network moved from the frontal lobe to the more extensive cortex as the disease progressed. We confirmed that the gray matter network might commence reorganization and show high resilience to network integrity damages in the early and preclinical stages of dementia, which is similar to previous studies (De Vogelaere et al., 2012). Thus, structural network properties can be a sensitive and reliable index to detect changes in the evolution of AD.

These findings are in line with previous studies reporting altered graph theoretical properties in these regions in AD-spectrum patients (Shah et al., 2018; Liu et al., 2020). Most of the network graph theoretical properties referring to the frontal lobe, medial temporal lobe, and subcortical structures–areas that play a role in perception, executive control, episodic memory, and understanding–have consistently been found to be affected across the development of AD (Geib et al., 2017; Danti et al., 2018; Luo et al., 2018). Furthermore, our study may reflect reorganization and high resilience to network integrity damage. Previous studies evaluating graph theoretical properties have described compensation at the level of hippocampal/parahippocampal regions and the frontal and occipital lobes (van Duinkerken et al., 2016; Li et al., 2018). In line with prior studies, in our analysis, network integrity was widely increased due to the compensation in specific nodes related to cognition. Despite differences in methodologies, compensation has been described in other neurological and psychiatric disorders, such as schizophrenia (Sapara et al., 2014) and early stages of Parkinson’s disease (Nonnekes et al., 2019). Increased global connectivity in the frontal lobe, hippocampus, and occipital areas has been previously reported for mild AD patients (Bai et al., 2011; De Vogelaere et al., 2012). Similarly, our study shows that the compensation appears in the medial temporal lobe at the brain regional level, while it gradually spread from the frontal lobe to the widely distributed throughout the cortex at the whole-brain network level with AD degenerative processes. However, there is no specific report about the potential mechanism revealing the patterns of this compensation within structural networks of AD-spectrum patients. In that sense, our findings present novel evidence of pathophysiological mechanisms in alterations within the gray matter network of AD-spectrum patients.

In addition, little is known about the pathological basis of structural network compensation (Jackson et al., 2019). The findings of impaired graph theoretical properties with reference to the frontal lobe, medial temporal lobe, and subcortical structures involved alterations affecting gray matter structures in the present AD spectrum patients, which is in line with previous studies reporting increased Aβ deposition and pathological tau accumulation in these regions in AD (Buckley et al., 2017). This study demonstrates the compensation related to cognitive impairments, which exists with a potential AD pathological basis behind them. Taken together, our findings and those from structural network studies suggest structural brain compensation in response to brain damage (Wook Yoo et al., 2015; Liu et al., 2020). However, the relationship between structural changes and disease progression remains controversial. Modifications in the cerebral structure could be integral mechanisms that reflect maladaptive changes promoting clinical dysfunction or maintaining optimal network functioning (Llufriu et al., 2017). Therefore, longitudinal studies are required to understand the positive or negative consequences of these compensatory brain changes.

Although our study attempted to provide a new perspective for understanding the aberrant structural network architecture and early identification in AD-spectrum patients, a few limitations still require future study. First, to explore the relationships among CSF pathology indicators, gray matter network graph theoretical properties, and cognitive function, we did not perform a correction for multiple comparisons. The present study was a preliminary exploration and this study, at least in part, revealed these interactions. Second, this study was cross-sectional, and no directionality or causal inferences were made. We still require large sample and longitudinal studies to further confirm these findings and to formulate a personalized evaluation system for disease progression in patients with cognitive impairment in the future. Third, the structural network parameters were calculated according to the binary adjacency matrix rather than the weighted network analysis. The latter analysis may provide additional findings in future studies. Fourth, the whole brain was divided coarsely into 90 regions based on the AAL template for structural network construction. The parcelation of the brain regions might influence the network properties and may result in various outcomes in the graph theoretical metrics. Different parcelation strategies are required to validate our findings.

## Conclusion

In summary, this study explored the differences in gray matter network properties by graph theory and revealed a reorganization mechanism of structural networks related to cognitive impairments and CSF pathological biomarkers in AD-spectrum patients. Our findings present novel evidence of compensatory mechanisms in gray matter networks of AD spectrum patients and highlight the potential for applying structural network metrics to monitor disease progression.

## Data Availability Statement

The raw data supporting the conclusions of this article will be made available by the authors, without undue reservation.

## Ethics Statement

The studies involving human participants were reviewed, approved by the ADNI database (http://adni.loni.usc.edu) as well as the written informed consents from the patients/participant.

## Author Contributions

FB, HZ, and YX designed the study and edited the manuscript. XS and HC analyzed the data and wrote the manuscript. HC, RQ, and PS validated the statistics. ADNI collected the data. All the authors contributed to the article and approved the submitted version.

## Conflict of Interest

The authors declare that the research was conducted in the absence of any commercial or financial relationships that could be construed as a potential conflict of interest.

## Footnotes

1 adni.loni.usc.edu

2 http://adni.loni.usc.edu/research/protocols/bios-pecimens-protocols/

3 http://adni.loni.usc.edu/

4 http://www.neuro.uni-jena.de/cat/

5 http://www.fil.ion.ucl.ac.uk/spm/soft-ware/spm12/

6 http://www.nitrc.org/projects/gretna/

7 https://github.com/bettytijms/Single_Subject_Grey_Matter_Networks
