# Supplementary material for: Brain Structural Network Compensation Is Associated With Cognitive Impairment and Alzheimer’s Disease Pathology
Source: Front Neurosci. 2021 Feb 25;15:630278. doi: 10.3389/fnins.2021.630278 (PMC7947929; doi:10.3389/fnins.2021.630278)
Supplement: Supplementary file 1 [file Table_1.DOCX]

**Supplemental Table 1.**

| **Brain areas and their abbreviations in the AAL-90 atlas** |
| --- |

| Regions | Abb. | Regions | Abb. |
| --- | --- | --- | --- |
| Precental gyrus | PreCG | Lingual gyrus | LING |
| Superior frontal gyrus, dorsolateral | SFGdor | Superior occipital gyrus | SOG |
| Superior frontal gyrus, orbital part | ORBsup | Middle occipital gyrus | MOG |
| Middle frontal gyrus | MFG | Inferior occipital gyrus | IOG |
| Middle frontal gyrus, orbital part | ORBmid | Fusiform gyrus | FFG |
| Inferior frontal gyrus, opercular part | IFGoperc | Postcentral gyrus | PoCG |
| Inferior frontal gyrus, triangular part | IFGtriang | Superior parietal gyrus | SPG |
| Inferior frontal gyrus, orbital part | ORBinf | Inferior parietal, but supramarginal and angular gyri | IPL |
| Rolandic operculum | ROL | Supramarginal gyrus | SMG |
| Supplementary motor area | SMA | Angular gyrus | ANG |
| Olfactory cortex | OLF | Precuneus | PCUN |
| Superior frontal gyrus, medial | SFGmed | Paracentral lobule | PCL |
| Superior frontal gyrus, medial orbital | ORBsupmed | Caudate nucleus | CAU |
| Gyrus rectus | REC | Lenticular nucleus putamen | PUT |
| Insula | INS | Lenticular nucleus, pallidum | PAL |
| Anterior cingulate and paracingulate gyri | ACG | Thalamus | THA |
| Median cingulate and paracingulate gyri | DCG | Heschl gyrus | HES |
| Posterior cingulate gyrus | PCG | Superior temporal gyrus | STG |
| Hippocampus | HIP | Temporal pole: superior temporal gyrus | TPOsup |
| Parahippocampal gyrus | PHG | Middle temporal gyrus | MTG |
| Amygdala | AMYG | Temporal pole: middle temporal gyrus | TPOmid |
| Calcarine ﬁssure and surrounding cortex | CAL | Inferior temporal gyrus | ITG |
| Cuneus | CUN |  |  |
