# Supplementary material for: Brain Structural Network Compensation Is Associated With Cognitive Impairment and Alzheimer’s Disease Pathology
Source: Front Neurosci. 2021 Feb 25;15:630278. doi: 10.3389/fnins.2021.630278 (PMC7947929; doi:10.3389/fnins.2021.630278)
Supplement: Supplementary file 2 [file Table_2.DOC]

**Supplemental Table 2.**

**Relationships between network metrics associated with neuropsycholohical performance in AD-spectrum patients**

| Group | network metrics |  | MMSE | MoCA | FAQ | CDRSB | ADAS13 | EcogSP Mem | EcogSP Lang | EcogSP Visspat | EcogSP Plan | EcogSP Organ | EcogSP Divatt | EcogSP Total |
| --- | --- | --- | --- | --- | --- | --- | --- | --- | --- | --- | --- | --- | --- | --- |
| HC | BC of PHG.R | r | -0.023 | -0.318 | 0.282 | 0.37 | 0.04 | 0.297 | 0.392 | 0.355 | 0.386 | 0.464 | 0.348 | 0.394 |
|  |  | p | 0.897 | 0.067 | 0.107 | 0.031* | 0.088 | 0.088 | 0.022* | 0.039* | 0.024* | 0.006** | 0.044* | 0.021* |
|  | DC of PHG.R | r | -0.092 | -0.248 | 0.352 | 0.424 | -0.009 | 0.296 | 0.305 | 0.492 | 0.436 | 0.484 | 0.491 | 0.4 |
|  |  | p | 0.606 | 0.157 | 0.041* | 0.012* | 0.089 | 0.089 | 0.08 | 0.003** | 0.01** | 0.004** | 0.003** | 0.019* |
|  | DC of AMYG.R | r | 0.022 | -0.177 | 0.36 | 0.388 | -0.117 | -0.122 | -0.037 | 0.196 | -0.074 | 0.039 | -0.225 | -0.162 |
|  |  | p | 0.901 | 0.317 | 0.037* | 0.023* | 0.493 | 0.493 | 0.837 | 0.267 | 0.678 | 0.825 | 0.202 | 0.36 |
|  | NE of PHG.R | r | -0.091 | -0.246 | 0.362 | 0.433 | 0.014 | 0.31 | 0.328 | 0.477 | 0.454 | 0.494 | 0.498 | 0.413 |
|  |  | p | 0.607 | 0.161 | 0.035* | 0.011* | 0.075 | 0.075 | 0.058 | 0.004** | 0.007** | 0.003** | 0.003** | 0.015* |
| MCI | BC of PHG.R | r | -0.04 | -0.08 | 0.301 | 0.295 | 0.103 | 0.207 | 0.199 | 0.223 | 0.326 | 0.159 | 0.122 | 0.227 |
|  |  | p | 0.741 | 0.511 | 0.011* | 0.013* | 0.398 | 0.086 | 0.099 | 0.063 | 0.006** | 0.187 | 0.313 | 0.059 |
|  | DC of PHG.R | r | -0.091 | -0.23 | 0.201 | 0.343 | 0.201 | 0.114 | 0.222 | 0.199 | 0.186 | 0.095 | 0.106 | 0.171 |
|  |  | p | 0.455 | 0.056 | 0.095 | 0.004** | 0.096 | 0.346 | 0.064 | 0.098 | 0.123 | 0.432 | 0.381 | 0.156 |
|  | DC of AMYG.R | r | -0.129 | -0.101 | -0.079 | -0.034 | 0.046 | -0.236 | -0.062 | -0.244 | -0.229 | -0.062 | -0.188 | -0.192 |
|  |  | p | 0.287 | 0.407 | 0.516 | 0.782 | 0.707 | 0.049* | 0.61 | 0.041* | 0.057 | 0.612 | 0.12 | 0.112 |
|  | NE of PHG.R | r | -0.056 | -0.231 | 0.199 | 0.286 | 0.204 | 0.132 | 0.248 | 0.149 | 0.209 | 0.077 | 0.121 | 0.172 |
|  |  | p | 0.647 | 0.055 | 0.098 | 0.016* | 0.09 | 0.278 | 0.038* | 0.217 | 0.082 | 0.528 | 0.32 | 0.155 |
| AD | BC of PHG.R | r | 0.088 | 0.06 | 0.061 | 0.192 | 0.111 | -0.008 | 0.172 | 0.334 | 0.157 | 0.012 | 0.118 | 0.209 |
|  |  | p | 0.589 | 0.714 | 0.707 | 0.235 | 0.496 | 0.96 | 0.29 | 0.035* | 0.334 | 0.942 | 0.469 | 0.197 |
|  | DC of PHG.R | r | -0.006 | -0.17 | 0.334 | 0.339 | 0.395 | 0.25 | 0.27 | 0.462 | 0.302 | 0.181 | 0.28 | 0.407 |
|  |  | p | 0.973 | 0.293 | 0.035* | 0.032* | 0.012* | 0.119 | 0.092 | 0.003** | 0.058 | 0.264 | 0.08 | 0.009** |
|  | DC of PHG.R | r | -0.126 | -0.206 | 0.335 | 0.271 | 0.23 | 0.286 | 0.307 | 0.384 | 0.395 | 0.021 | 0.075 | 0.389 |
|  |  | p | 0.438 | 0.202 | 0.034* | 0.091 | 0.154 | 0.074 | 0.054 | 0.014* | 0.012* | 0.898 | 0.646 | 0.013* |
|  | NE of PHG.R | r | -0.069 | -0.185 | 0.342 | 0.362 | 0.364 | 0.292 | 0.289 | 0.511 | 0.336 | 0.208 | 0.328 | 0.446 |
|  |  | p | 0.67 | 0.253 | 0.031* | 0.022* | 0.021* | 0.067 | 0.071 | 0.001** | 0.034* | 0.197 | 0.039* | 0.004** |
| *P<0.05, **P<0.01 indicates an uncorrected relevant analysis | | | | | | | | | | | | | | |
| Abbreviations: HC, healthy controls; MCI, mild cognitive impairment;AD,Alzheimer's disease; BC, Betweenness Centrality; DC, Degree Centrality; NE, Nodal Efficiency; PHG.R, right parahippocampal gyrus; AMYG.R, right amygdala; MMSE, mini mental state examination; MoCA, Montreal Cognitive Assessment; FAQ, Functional Activities Questionnaire; CDRSB, Clinical Dementia Rating Sum of Boxes; ADAS13, Alzheimer's Disease Assesment Scale; EcogSP, Everyday Cognition by the patient's study; Mem, Memory; Lang, Language; Visspat, Visuospatial; Plan, Planning; Organ, Organization; Divatt, Divided Attention. | | | | | | | | | | | | | | |
|  |  |  |  |  |  |  |  |  |  |  |  |  |  |  |
