# Supplementary material for: Brain Structural Network Compensation Is Associated With Cognitive Impairment and Alzheimer’s Disease Pathology
Source: Front Neurosci. 2021 Feb 25;15:630278. doi: 10.3389/fnins.2021.630278 (PMC7947929; doi:10.3389/fnins.2021.630278)
Supplement: Supplementary file 3 [file Table_3.DOC]

**Supplemental Table 3.**

**Relationships between altered edges properties and neuropsycholohical performance in MCI patients**

| Interregional similarity |  | MMSE | MoCA | FAQ | CDRSB | ADAS13 | EcogSP Mem | EcogSP Lang | EcogSP Vission | EcogSP Plan | EcogSP Orgnize | EcogSP Diva | EcogSP Total |
| --- | --- | --- | --- | --- | --- | --- | --- | --- | --- | --- | --- | --- | --- |
| SFGmed.R - PreCG.L | r | 0.109 | -0.078 | -0.028 | -0.104 | -0.008 | -0.017 | -0.021 | -0.039 | 0.1 | -0.075 | 0.158 | -0.015 |
|  | *p* | 0.369 | 0.519 | 0.82 | 0.391 | 0.948 | 0.892 | 0.862 | 0.749 | 0.412 | 0.535 | 0.191 | 0.9 |
| SFGmed.R - MFG.R | r | 0.356 | 0.208 | 0.027 | -0.162 | 0.019 | 0.199 | 0.163 | 0.174 | 0.154 | 0.066 | 0.154 | 0.181 |
|  | *p* | 0.002** | 0.084 | 0.822 | 0.179 | 0.878 | 0.099 | 0.178 | 0.15 | 0.204 | 0.589 | 0.204 | 0.135 |
| SFGmed.R - ORBsupmed.L | r | -0.277 | -0.089 | 0.15 | 0.232 | 0.136 | 0.19 | 0.012 | 0.153 | -0.055 | 0.084 | 0.086 | 0.147 |
|  | *p* | 0.02* | 0.466 | 0.215 | 0.053 | 0.262 | 0.116 | 0.925 | 0.207 | 0.648 | 0.491 | 0.479 | 0.224 |
| SFGmed.R - ORBsupmed.R | r | -0.202 | 0.066 | 0.005 | 0.003 | 0.005 | 0.035 | -0.044 | -0.071 | 0.007 | 0.067 | 0.013 | 0.018 |
|  | *p* | 0.094 | 0.589 | 0.967 | 0.979 | 0.97 | 0.774 | 0.715 | 0.558 | 0.951 | 0.581 | 0.912 | 0.88 |
| SFGmed.R - REC.L | r | -0.141 | -0.053 | -0.058 | 0.127 | 0.052 | 0.06 | 0.07 | -0.141 | 0.033 | -0.006 | -0.026 | 0.032 |
|  | *p* | 0.245 | 0.665 | 0.635 | 0.296 | 0.667 | 0.623 | 0.563 | 0.245 | 0.785 | 0.958 | 0.829 | 0.79 |
| SFGmed.R - PHG.L | r | -0.158 | -0.134 | 0.057 | 0.013 | 0.179 | -0.002 | 0.117 | -0.076 | -0.08 | -0.113 | -0.141 | -0.033 |
|  | *p* | 0.192 | 0.27 | 0.638 | 0.913 | 0.139 | 0.989 | 0.333 | 0.534 | 0.508 | 0.353 | 0.245 | 0.789 |
| SFGmed.R - CUN.L | r | 0.169 | 0.106 | -0.059 | -0.046 | 0.003 | -0.123 | -0.003 | -0.225 | -0.168 | -0.044 | -0.007 | -0.117 |
|  | *p* | 0.163 | 0.382 | 0.626 | 0.704 | 0.982 | 0.309 | 0.981 | 0.061 | 0.166 | 0.718 | 0.952 | 0.334 |
| SFGmed.R - FFG.R | r | -0.012 | -0.039 | -0.1 | -0.11 | -0.019 | -0.182 | -0.132 | -0.224 | 0.308 | -0.046 | -0.221 | -0.205 |
|  | *p* | 0.921 | 0.747 | 0.408 | 0.367 | 0.875 | 0.132 | 0.277 | 0.062 | 0.009** | 0.707 | 0.066 | 0.088 |
| **P*<0.05, ***P*<0.01 indicates an uncorrected relevant analysis | | | | | | | | | | | | | |
| Abbreviations: MMSE, mini mental state examination; MoCA, Montreal Cognitive Assessment; FAQ, Functional Activities Questionnaire; CDRSB, Clinical Dementia Rating Sum of Boxes; ADAS13, Alzheimer's Disease Assesment Scale; EcogSP, Everyday Cognition by the patient's study; Mem, Memory; Lang, Language; Visspat, Visuospatial; Plan, Planning; Organ, Organization; Divatt, Divided Attention; PreCG.L, left precental gyrus; MFG.R, right middle frontal gyrus; SFGmed.R, right superior frontal gyrus-medial part; ORBsupmed.L, left superior frontal gyrus-medial orbital part; ORBsupmed.R, right superior frontal gyrus-medial orbital part; REC.L, left gyrus rectus; PHG.L, left parahippocampal gyrus; CUN.L, left cuneus; FFG.R, right fusiform gyrus. | | | | | | | | | | | | | |
|  |  |  |  |  |  |  |  |  |  |  |  |  |  |
