# Supplementary material for: Brain Structural Network Compensation Is Associated With Cognitive Impairment and Alzheimer’s Disease Pathology
Source: Front Neurosci. 2021 Feb 25;15:630278. doi: 10.3389/fnins.2021.630278 (PMC7947929; doi:10.3389/fnins.2021.630278)
Supplement: Supplementary file 4 [file Table_4.DOC]

**Supplemental Table 4.**

**Relationships between altered edges properties and neuropsycholohical performance in AD**

| Interregional similarity |  | MMSE | MoCA | FAQ | CDRSB | ADAS13 | EcogSP Mem | EcogSP Lang | EcogSP Vission | EcogSP Plan | EcogSP Orgnize | EcogSP Diva | EcogSP Total |
| --- | --- | --- | --- | --- | --- | --- | --- | --- | --- | --- | --- | --- | --- |
| SFGmed.R - PreCG.L | r | -0.173 | -0.11 | 0.161 | 0.08 | 0.086 | -0.067 | -0.176 | 0.03 | 0.06 | -0.151 | -0.034 | -0.053 |
|  | *p* | 0.287 | 0.498 | 0.321 | 0.626 | 0.599 | 0.681 | 0.278 | 0.857 | 0.715 | 0.352 | 0.833 | 0.745 |
| SFGmed.R - PreCG.R | r | -0.396 | -0.35 | 0.385 | 0.278 | 0.144 | 0.187 | 0.198 | 0.146 | 0.206 | 0.01 | 0.014 | 0.196 |
|  | *p* | 0.011* | 0.027* | 0.014* | 0.082 | 0.374 | 0.248 | 0.221 | 0.369 | 0.202 | 0.95 | 0.93 | 0.226 |
| SFGmed.R - PoCG_R | r | -0.165 | -0.395 | 0.295 | 0.315 | 0.084 | -0.193 | -0.063 | 0.046 | -0.067 | -0.03 | -0.191 | -0.098 |
|  | *p* | 0.309 | 0.012* | 0.064 | 0.048* | 0.607 | 0.234 | 0.698 | 0.78 | 0.679 | 0.855 | 0.238 | 0.547 |
| SFGmed.R - THA.R | r | -0.16 | -0.224 | 0.099 | 0.092 | 0.147 | -0.068 | -0.104 | 0.037 | -0.002 | 0.155 | -0.219 | -0.005 |
|  | *p* | 0.323 | 0.166 | 0.542 | 0.574 | 0.366 | 0.677 | 0.522 | 0.82 | 0.991 | 0.34 | 0.176 | 0.977 |
| OLF.R - AMYG.R | r | -0.03 | -0.113 | 0.205 | 0.132 | 0.135 | 0.112 | 0.022 | 0.236 | 0.272 | -0.05 | 0.003 | 0.126 |
|  | *p* | 0.857 | 0.488 | 0.203 | 0.417 | 0.405 | 0.49 | 0.892 | 0.142 | 0.089 | 0.759 | 0.987 | 0.44 |
| OLF.R - THA.R | r | -0.124 | -0.258 | 0.008 | 0.236 | 0.26 | -0.051 | -0.028 | 0.048 | 0.003 | 0.021 | -0.137 | -0.057 |
|  | *p* | 0.445 | 0.108 | 0.961 | 0.142 | 0.105 | 0.756 | 0.865 | 0.77 | 0.986 | 0.899 | 0.399 | 0.729 |
| ACG.L - THA.R | r | -0.15 | -0.349 | 0.135 | 0.263 | 0.354 | 0.04 | 0.035 | 0.16 | 0.103 | 0.127 | -0.083 | 0.067 |
|  | *p* | 0.355 | 0.027* | 0.405 | 0.102 | 0.025* | 0.807 | 0.828 | 0.324 | 0.527 | 0.435 | 0.611 | 0.679 |
| **P*<0.05, ***P*<0.01 indicates an uncorrected relevant analysis | | | | | | | | | | | | | |
| Abbreviations: AD, Alzheimer’s disease; MMSE, mini mental state examination;MoCA, Montreal Cognitive Assessment;FAQ,Functional Activities Questionnaire; CDRSB, Clinical Dementia Rating Sum of Boxes; ADAS13, Alzheimer's Disease Assesment Scale; EcogSP, Everyday Cognition by the patient's study; Mem, Memory; Lang, Language; Visspat, Visuospatial; Plan, Planning; Organ, Organization; Divatt, Divided Attention; PreCG.L, left precental gyrus; PreCG.R, right precental gyrus; OLF.R, right olfactory cortex; SFGmed.R, right superior frontal gyrus-medial part; ACG.L, left anterior cingulate and paracingulate gyri; AMYG.R, right amygdala; PoCG.R, right postcentral gyrus; THA.R, right thalamus. | | | | | | | | | | | | | |
|  |  |  |  |  |  |  |  |  |  |  |  |  |  |
