# Supplementary material for: Brain Structural Network Compensation Is Associated With Cognitive Impairment and Alzheimer’s Disease Pathology
Source: Front Neurosci. 2021 Feb 25;15:630278. doi: 10.3389/fnins.2021.630278 (PMC7947929; doi:10.3389/fnins.2021.630278)
Supplement: Supplementary file 5 [file Table_5.DOC]

Lumbar puncture and cerebrospinal fluid (CSF) sample preparation were performed as described in the ADNI manual (<http://adni.loni.usc.edu/research/protocols/biospecimens-protocols/>). Aβ1-42, t-tau, and p-tau were measured in each of the 416 CSF ADNI baseline aliquots using the multiplex xMAP Luminex platform (Luminex Corp, Austin, TX) with Innogenetics (INNO-BIA AlzBio3; Ghent, Belgium; for research use-only reagents) immunoassay kit-based reagents. Full details of this combination of immunoassay reagents and analytical platform are provided elsewhere. In brief, Innogenetics kit reagents included well-characterized capture monoclonal antibodies specific for Aβ1-42 (4D7A3), t-tau (AT120), and p-tau (AT270), each chemically bonded to unique sets of color-coded beads, and analyte-specific detector antibodies (HT7, 3D6). Calibration curves were produced for each biomarker using aqueous buffered solutions that contained the combination of three biomarkers at concentrations. Before performing these analyses of the ADNI and the independent autopsy-based CSF samples in the ADNI University of Pennsylvania ADNI Biomarker Core laboratory, an interlaboratory study was conducted to qualify the performance conditions, including all major variables that can affect the test results, for the immunoassay reagents and analytical platform.

The clustering coefficient C_i_ of a node v_i_ is defined as the number of edges k_j_ between its direct neighbors (denoted by subgraph G_i_) divided by the total number of all possible edges K_Gi_ in Gi, which can be defined as:

*G*_i_ is the subgraph consisted of the nearest neighbors of node *i*.

The global clustering coefficient (Cp) of the network is the average clustering coefficient over all N nodes$Cp\left(G\right)=\frac{1}{N}\sum_{i\in G} C(i)$. It is calculated as：

where N is the number of nodes in the network G, and C(i) is defined as the number of edges between its direct neighbors divided by the total number of all possible edges.

Nodal clustering coefficient characterizes the possibility of neighbor node connected to each other. It is calculated as:

*E_i_* is the actual number of edges between node i connected to all the other nodes.

Characteristic path length is an indicator of the whole network connectedness and can quantify the parallel information propagation ability, which can be calculated as:

*d_ij_* is the characteristic path length between nodes *i* and nodes *j*.

Nodal characteristic path length measures the routing efficiency or mean distance between any two nodes in the network , which can be calculated as：

*d_ij_* means the shortest path length between node i and node j in the network.
